# Improvement Science Meets Improvement Scholarship: Reframing Research for Better Healthcare

**DOI:** 10.1007/s10728-017-0354-6

**Published:** 2017-12-21

**Authors:** Alan Cribb

**Affiliations:** Centre for Public Policy Research, King’s College London, Waterloo Bridge Wing FWB, London, SE1 9NH UK

**Keywords:** Quality improvement, Improvement science, Normativity, Moral seriousness

## Abstract

In this editorial essay I explore the possibilities of ‘improvement scholarship’ in order to set the scene for the theme of, and the other papers in, this issue. I contrast a narrow conception of quality improvement (QI) research with a much broader and more inclusive conception, arguing that we should greatly extend the existing dialogue between ‘problem-solving’ and ‘critical’ currents in improvement research. I have in mind the potential for building a much larger conversation between those people in ‘improvement science’ who are expressly concerned with tackling the problems facing healthcare and the wider group of colleagues who are engaged in health-related scholarship but who do not see themselves as particularly interested in quality improvement, indeed who may be critical of the language or concerns of QI. As one contribution to that conversation I suggest that that the increasing emphasis on theory and rigour in improvement research should include more focus on normative theory and rigour. The remaining papers in the issue are introduced including the various ways in which they handle the ‘implicit normativity’ of QI research and practice, and the linked theme of combining relatively ‘tidy’ and potentially ‘unruly’ forms of knowledge.

To what practical ends might health-related research—including scholarship in the humanities and critical social sciences that is often not conceived of as ‘problem-solving’ or instrumentally oriented—be put? In particular what are the prospects of fostering a productive conversation between such scholarship and those who have a very immediate concern with improving the quality of healthcare? That is the theme of this issue of Health Care Analysis.

In what follows I am interested in imagining the ‘coming together’ of two groups of scholars. The first group are those who explicitly see themselves as practising ‘improvement research’ (or improvement science, implementation science or similar)—that is, research in healthcare quality improvement that is explicitly aimed at improving health services. The second, much more diffuse, group are all of those humanities and social science scholars who are interested in healthcare and health services and who have diverse sets of aims that may or may not include some sense that their work is aimed at improving things. The ‘coming together’ I am imagining here is simply an introductory meeting—I would like to imagine that somewhere down the line these groups are much closer together; but that is rushing ahead.

I am not invoking ‘critical currents’ of scholarship on the grounds that they can be defended as valuable in themselves—although I accept that. Rather the key conversation I have in mind here can be seen as one between two currents of moral seriousness—currents which exist both outside and inside improvement research. On the one hand there is the ethical imperative to address the inadequacies of health policies and services, including the substantial harms that are caused by healthcare deficiencies. On the other hand there is the less pressing but still practically relevant sceptical task of asking how well equipped we are to ‘improve’ things—how far we know what we are talking about and doing when we seek to be useful in this way. The debate between these two forms of moral seriousness is alive and well within the quality improvement research community but the question is whether and how it might be usefully extended. As I will go on to argue QI is an inherently normative field and its development, I suggest, depends upon harnessing both currents of moral seriousness in combination.

## Healthcare Quality Improvement

In the next section I will entertain ideas about extended conceptions of ‘quality improvement’ but it is worth signalling the huge importance of the field that is conventionally described in these terms. Quality improvement (QI) research and practice sits at the intersection of numerous fields including healthcare professionalism, health service design, health service management and public health. It focuses on the underpinnings of good quality healthcare, with an emphasis on the identification and rectification of shortfalls in care delivery, looking beyond lapses in individual judgement towards the many ways in which the organisation, climates, routines and norms of health services can either produce or fail to prevent various kinds of harms (and sometimes ‘wrongs’) to patient populations. Although it has developed into an intellectually rich and eclectic field it owes much of its core to industrial models of QI and to the underlying logic that good quality care depends upon a ‘systems’ approach. That is, good quality is not just about educating practitioners well or creating access to the right ‘evidence-based’ treatments—not that these things are at all straightforward. But unless these things are also embedded and activated within optimal conditions (e.g. good appointment systems, established interprofessional working, reliable storage and communication of clinical data, patient engagement, safety checklists, cleaning practices and so on) then poor care can and often will result. The gravity of these matters is highlighted in public consciousness from time to time by high profile and dramatically reported scandals but, in fact, is long established and well understood within the QI community through countless sober analyses of routine quality failures. QI now has an established place high on the healthcare policy agenda, is supported by international and national organisations and agencies, and is a fast growing area of practice. QI practice and research are closely interlinked. QI practices should be research-informed and depend, at the very least, on processes of evaluation, measurement and reflection, and QI research is inherently applied research—geared to being used to inform policy and practice.

Good quality healthcare is typically seen in multi-dimensional terms. For example, the highly influential account from the US Institute of Medicine characterises good quality care as care that is “safe, effective, patient-centred, timely, efficient, and equitable” with each of these quality ‘aims’ being further elaborated [10]. This broad conception of quality allows for the possibility of QI being about the pursuit of increasing ‘excellence’, i.e. with would-be improvers striving to further strengthen, refine and ‘perfect’ forms of provision that are already clearly above any sensible threshold of good care. However, as I have already indicated, in reality the emphasis in the field is typically about pushing standards upwards towards a threshold of ‘good care’ and, in particular, to seek to address cases of (and especially systematic tendencies towards) bad quality care. This emphasis is not surprising and seems practically and ethically defensible. It also reflects the tenor of the document in which the IOM’s account of quality is set out which begins “Americans should be able to count on receiving care that meets their needs and is based on the best scien-tific knowledge—yet there is strong evidence that this frequently is not the case. Healthcare harms patients too frequently and routinely fails to deliver its potential benefits. Indeed, between the health care that we now have and the healthcare that we could have lies not just a gap, but a chasm.” [10].

QI research in health service contexts has some clear resonances with public health research lenses (I will return to this). For example, it needs to capture and reflect the incidence and prevalence, and the determinants of, poor quality. But it also needs to understand the effectiveness of QI interventions and the factors that influence various degrees and kinds of effectiveness. And—as with public health—there can be a significant mismatch between the robustness of research in these two domains. Specifically it is possible that we might have very good grounds for identifying an area where healthcare “routinely fails to deliver its potential benefits” (based for example, on studies of variations between service units in relation to treatment outcomes—both clinically and patient-defined—in matched patient groups); but we may have comparatively little insight into what mechanisms hold promise for improving the outcomes of the poorer performing units. In short we may have convincing evidence for asserting that ‘there is a quality shortfall here and something needs to be done about it’, but no firm basis for recommending that was is done is, specifically, ‘x’ or ‘y’, or for advocating any particular ‘something’. Of course it is often very easy—and therefore tempting—to point to settings where ‘x’ or ‘y’ seems to be associated with better quality. But the notorious problems of understanding relatively open-ended systems, including of taking into account ‘context’ in making generalisations, should encourage a degree of caution. Here is one place where the tension between the two aspects of moral seriousness can become evident—we may feel obliged to address a quality shortfall but worry lest we waste our effort or even make things worse.

## Making Things Better

Beyond the official world of QI there are, of course, all kinds of ways in which people might be pursuing improvement-relevant scholarship. Presumably anyone working in health services research, and most anyone who is engaged in health-related applied humanities and social sciences scholarship, has an interest in ‘making things better’ in some sense. Even in those cases of scholarship where the stress is heavily on ‘truth’ rather than ‘use’ there is, at a foundational level, a relationship between knowledge and practical interests. There are—in Habermas’ terms—‘knowledge constitutive interests’; in other words, even when research is not geared towards discovering technical solutions for problems it can be motivated and shaped by other practical ends, such as ‘mutual understanding’ and/or emancipatory ideals [16]. This, along with the sceptical or deconstructive potential of critical scholarship already mentioned, has the implication that even where scholars are not involved in (and may indeed be hostile to the business of) formulating recommendations for policy makers or practitioners their work may have potential to contribute to ‘quality’ or ‘improvement’ in some sense.

The range of scholarship that can be viewed as relevant to QI is vast (without including, for now, those scholarly approaches that centre on critique, including deconstruction). The literature expressly oriented to QI already acknowledges the importance of ‘governmental’, ‘economic’, and ‘professional’ ways of improving quality as well as the ‘industrial’ models highlighted in the previous section. That is to say if we want to improve quality we could look beyond specific ‘QI approaches’ towards such things as legislation, regulation, performance management, or reforming models of funding, competition or coordinated commissioning, or towards professional education, peer review or collaboration, and so on [13]. Of course the vast majority of scholarly work on these themes is not conducted under the explicit label of QI but labelling is not a guide to relevance or potential usefulness. It is a reasonable bet that most scholars working in these diverse areas have some more or less direct interest in the effectiveness and efficiency of healthcare—in how healthcare might be more successfully, and less wastefully, paid for, organised or delivered. All of this without, necessarily, any use of the words ‘quality’ or ‘improvement’.

The same applies if we shift from a macro-framing to a micro-framing. The official lexicon of QI features ‘patient-centredness’ as a dimension of quality. This notion alone—and more so when it is conjoined to the quality dimension of ‘equity’—points towards a complex, multi-dimensional set of concerns: is people’s suffering acknowledged and attended to; more broadly is healthcare experienced as valuable; are individuals and communities treated with respect within healthcare processes; how far is healthcare built upon collaborative relations or co-produced; how is power distributed, how are people, in all of their diversity, and their voices and identities included or excluded, and so forth? Once again there is a vast array of different types of scholarship, from many different disciplines, that have relevance here. Those studying, to take a few random examples, autobiography, emotional labour, critical race theory, phenomenology, political theory, the ethics of trust, and many other themes have something to contribute here even if they have never heard of QI.

Perhaps the risk here—in what might playfully be called the unwitting extended improvement research community—is the opposite of the one mentioned in the previous section. The risk within QI research is that people may be too ready to ‘prescribe for improvement’, whereas by contrast those doing more loosely related ‘improvement-relevant scholarship’ may be very slow to make, and hesitant about making, recommendations even when they might be in a position to do so. This is background against which I believe it makes sense to call for richer and fuller conversations between the two currents of moral seriousness that I have crudely summarised in this contrast between overeagerness and reluctance.

## Between Problem-Solving and Critique: Expanding the Dialogue

The conversation I am envisaging here is no more than a continuation and expansion of an ongoing series of dialogues that are already established within improvement research. The field of ‘improvement science’ came about partly because people wanted to pursue the kinds of rigour that are expected in clinical research. How can we expect clinicians to be able to cite persuasive evidence in support of their individual clinical interventions but not demand something closely analogous when those same clinicians, or their colleagues, prescribe a ‘quality enhancing’ intervention to a whole service? But this broadly ‘technicist’ instinct has been accompanied by more sceptical and liberal instincts too. For a start the search for rigour naturally leads to a questioning of specific approaches or models of QI—such as PDSA cycles or Lean, Six Sigma etc. [4]. Are these, in specific cases, ‘fit for purpose’, sufficiently specified, applied with adequate fidelity and so on? Closely interwoven with this kind of questioning is an acknowledgement of some of the challenges of capturing the complexity of human action within institutional, cultural and policy systems. Because improvement research draws upon a range of social sciences it can access and make use of both ‘problem-solving’ and ‘critical’ theoretical resources and dispositions—sometimes aiming for useful accounts of what can be done, sometimes problematising simplistic ‘technicist’ formulae that fail to capture the density and slipperiness of the social world.

In other words with the advent of ‘improvement science’ there was a deliberate move beyond relatively narrow and pragmatic, often technocratic or ‘implementation-focussed’, approaches to healthcare improvement towards models of rigorous multi-disciplinary research that seek to take seriously, and where possible combine, the sometimes conflicting demands both of local practical wisdom and learning, and more ‘publishable’ and ‘generalisable’ knowledge [14]. Raising the epistemological demands and stakes in this way inevitably produces a field of ‘improvement research’ that strives to be both social science literate and critically reflexive. Two linked manifestations of this trend are (i) an increased interest in the kind of cross-fertilisation I am advocating here—cross-fertilisation between the core concerns of improvement research and broader traditions of social sciences and humanities; and (ii) a healthy sceptical current within improvement research—highlighting the potential shortcomings of QI work (and thereby some of the obstacles that need to be overcome if QI is to be more robust and effective). I will say a little more about each of these in turn.

An excellent model of explicit attention to scholarly cross-fertilisation is provided by the Sociology of Health and Illness monograph edited by Davina Allen and colleagues [1]. In it the editors contrast what they call the ‘orthodox paradigm’ of research on quality and safety with a ‘sociological paradigm’. The former roughly coincides with what I have previously characterised as an industrial-technocratic approach, which they summarise as drawing heavily on specific strands of behavioural sciences such as human factors research and ergonomics. By contrast the sociological paradigm is broader and more diffuse. Their argument is that sociological perspectives—although they have already made a significant contribution to improvement research—have much more potential than has been hitherto recognised. The further contributions they have in mind are both ‘complementary’ and a ‘critical’ ones. For example in one of their overview articles Justin Waring and the other editors show the central relevance and practical import—for healthcare quality and safety—of the tradition of work on the sociology of medical knowledge from Illich’s classic studies of ‘iatrogenesis’ onwards [20]. This body of work—which of course is far too rich to summarise here—illuminates the nature of professional and institutional power and how such power works through the way things are defined, assumptions about what is salient, about ‘who does what’ and how authority and responsibility are distributed and so forth. These insights alone are enough to suggest the crucial importance of looking beneath overt phenomena and drawing upon broader and more foundational lenses if we are to achieve a fuller understanding of, and repertoire for approaching, healthcare quality and safety. The insights provided by the sociology of medical knowledge can be multiplied by looking across and drawing upon a range of sociological themes and perspectives—this is because there are many and various ways in which discourses and practices related to healthcare quality and quality improvement actions (and omissions) are socially embedded:

“Viewed from a sociological perspective, a host of salient mediating factors are neglected or poorly theorised within the orthodox view, such as the social structuring of activities and practice; the interactional character of care; social boundaries; organisational and professional cultures; the power dynamics of care delivery; and the regulatory, political and institutional contexts of care. The failure to sufficiently consider these wider social, cultural and institutional factors might explain why quality improvement interventions are challenging to implement or why successes made in one setting are difficult to replicate.” [20].

Parallel concerns about the possible limitations of healthcare QI have emerged across established improvement research literature. Andrew Smaggus and Mark Goldszmidt [19] refer, as have others, to the dangers of ‘Cargo Cult QI’—i.e., QI that has the surface appearance of ‘science’ but fails to embody appropriate methodologies underneath this surface. Referring to the growth in the policy importance of QI and the increasing involvement of clinicians in QI efforts [18] they argue that “the pressure to pursue quality improvement (QI) initiatives may presently exceed our expertise for achieving them successfully….. While these developments are ostensibly positive, it is worth considering whether they have produced a superficial approach to quality and safety. It may be that we are incentivising efforts that quickly create the impression of pursuing quality (for administrators, department heads, and regulatory bodies), without adequately attending to the more arduous efforts required for developing expertise regarding the concepts, mechanisms and essential elements that create meaningful improvement.” [19] Across the improvement research literature more broadly there is a sense of innovation and of emerging approaches and paradigms but this is sometimes accompanied by a sense that there is a constant associated risk of the field ‘running before it is walking’ and of paradigms and models—including from core feeder disciplines—being adopted but not always fully understood [17].

The shortcomings of much current QI—the grounds for scepticism—along with possible ways forward, have been brilliantly summed up in the appositely titled paper “Does quality improvement improve quality by Mary Dixon-Woods and Graham Martin [6]. Their analysis crystallises the limitations of much QI work. These include the frequency with which QI is thought about and enacted in short-term and small-scale ways—underpinned by an assumption that specific ‘interventions’ operate like ‘magic bullets’. This tendency for the field to operate through multiple discrete projects with a heavy emphasis on innovation rather than replication—what they call ‘projectness’—reflects very significant theoretical and strategic deficits. At a theoretical level the underlying ‘magic bullet’ model fails to recognise the ways in which the effectiveness of QI initiatives—or of any action in a social field for that matter—need to be seen as a product of the interactions between initiative and context (such that neat distinctions here, and especially hasty and tidy assumptions about causation, must be problematised). At a strategic level ‘projectness’ is a potential liability because it fails to consider how interventions may have adverse effects on one another, or may have benefits at one level (e.g. within a unit) but cause problems at another (e.g. at an institutional or system level). These key insights lead to recommendations for greater coordination of effort, including a focus on curating of learning, capacity building and sector-level thinking drawing upon “expertise from multiple disciplines”.

## Reframing Research for Improvement?

What these emerging literatures amount to—whether they as appear as conversations *with* improvement research or *within* improvement research—are calls for reframing QI research agendas and approaches. There are a number of aspects to this reframing, which I will loosely classify as spatio-temporal, conceptual and normative—which, separately and in combination, suggest new methodological emphases or directions. The literature I have pointed towards encompasses each of these three aspects but I would suggest that the potential importance of normative reframing has not yet been sufficiently emphasised.

In plain terms what is at stake is ‘problem definition’. If we are interested in improving things where should we be looking, what combination of agents and mechanisms should we include or concentrate upon, and how should we assess progress? Spatio-temporal aspects of reframing include a preparedness (a) to think in terms of the challenges of working at, and across, micro, meso and macro levels, and not least the ways in which these levels are both causally and constitutively interconnected and (b) to adopt the same approach to time frames—seeing the short-term in the context of longer, cumulative processes. This expansion of vision is closely interwoven with potential for many forms of conceptual reframing. For example, Dixon-Woods and Martin’s call for a sector-wide perspective and long-term organisational learning necessitates a change of conceptual lenses. Again there are clear parallels here with the introduction of public health lenses into health service contexts—because, for example, structural, population and prevention-orientations bring and need different discourses and terms of art. More broadly the importance of conceptual reframing is very well illustrated and exemplified in the above mentioned work on the ‘sociological paradigm’ of improvement research. This work both questions the relatively untheorised use, within ‘orthodox’ improvement research, of concepts like ‘culture’ or ‘system’ and shows the potential for very many sociological concepts and frameworks to re-work and enrich the field.

The normative aspects of improvement research, and the scope for normative reframing, should not really be separated out from the spatio-temporal and conceptual aspects. But it is arguably worth doing so to show their relative neglect. They are certainly present in the work I have briefly reviewed here [2]. One of the main themes of the Allen et al. sociology collection [1] is the importance of political factors in QI—both large scale effects of power and such things as the value systems of professionals (whose norms and identities can be threatened by QI efforts). Most notably the sociological traditions of work mentioned earlier that have sprung from concerns about iatrogenesis have helped elaborate notions of ‘harm’, and the origins and consequences of forms of harm, in ways that have now entered mainstream thinking about such things as over diagnosis and ‘too much medicine’. Another explicit normative contribution is exemplified, in the same sociology collection, by a paper in which Dixon Woods and colleagues analyse the ethics of QI and particularly assumptions made about the distribution of accountability, and dilemmas concerning the assignment of responsibility to individual agents [3]. However, for the most part, debates about values and norms appear relatively marginal in both QI and associated research communities. This is especially striking given that ideas like ‘quality’ and ‘improvement’ are such conspicuously value-laden and ‘contested’ concepts [9].

As I have already indicated the dominant tone of QI has tended to be technicist—assuming a means-end rationality and with ends operationally defined. Of course this itself embodies some important values—it supports a focussing of effort, enables measurement of effectiveness (which is, rightly, often cited as a central plank of professional accountability), facilitates communication and transparency and so on. However this emphasis also serves to marginalise, or even positively obscure, very many value questions, including some fundamentally important ones.

Some of these questions fall into the category of ‘QI ethics’. But with a few exceptions ‘QI ethics’ has tended to be discussed in limited terms—as something parallel to, but distinctive from, healthcare research ethics (in other words has concentrated on identifying suitable approaches to the governance of QI mindful of the risks as well as benefits of QI interventions [8, 12]. This is a very important theme but there are other normative questions central to QI that are much less discussed. In a nutshell these concern the range of ‘goods’ that are, and ought to be, embodied in QI discourses and practices. For example, alongside debates about potentially relevant ‘harms’ to avoid, there are fundamental questions about underlying conceptions of healthcare purposes—these can be approached by analysing assumptions about concepts like ‘quality’ and ‘improvement’ but they have root and branch implications. To what extent, for instance, should the effectiveness of healthcare be defined in terms of its success at ‘disease management’ and to what extent by its contribution to enabling people to pursue lives that they value? [7] Similarly foundational questions can be asked about the processes of QI. For instance, how can professional virtues including the ‘QI virtues’ [11, 15] be underpinned by the design of QI programmes, and how might these be undermined by approaches that are heavily managerialist or otherwise narrowly prescribed? These matters link in with many other questions, including some briefly alluded to above about the structure/agency relationship and defensible allocations of responsibility. Taken together these foundational questions about purposes and processes can be used to problematise the whole enterprise of QI (in ways that resonate with some of the varieties of scepticism rehearsed above) but also used constructively to carefully unpick specific approaches, themes and cases—a huge and hugely varied agenda. It seems that the ever increasing emphasis on the theoretical bases of, and rigour in, improvement research needs to be stretched to encompass an interest in normative theory and rigour.

To contemplate the possibilities for spatio-temporally, conceptually and normatively reframing QI research is inevitably to invite new methodological and interdisciplinary conversations—to ask how should we think about QI and what kinds of perspectives might make what kinds of contributions. However, before hastily assuming that this is a recipe for a completely ‘open house’ it is important to bear in mind the distinction with which I began—between scholarship that is directly aimed at improving healthcare and scholarship that may have relevance to this goal but is produced with other aims. This is not a clear-cut distinction but it is an important one. Someone who is working for healthcare improvement—whether as a practitioner or as a researcher—has a different job than someone who is ‘producing knowledge’ with other ends in mind. As we move along the translational spectrum we are subject to different intellectual and ethical challenges and constraints [5, 21]. In simple terms if I want to claim that I am doing ‘improvement research’ then I need to be ready to be accountable (to policy makers, practitioners and other stakeholders including publics) both in terms of ensuring that what I am offering them is accessible and ‘applicable’—feasible and practicable and not just ‘applied’ in name—and to be ready to take my share of responsibility for the risks entailed by whatever actions are underpinned by my advice. For this reason, as well as possibly just because of intellectual humility, many researchers in applied humanities and social sciences—including even those of a ‘problem-solving’ disposition—may be reluctant to see what they do as falling within the category of improvement research. They may instead prefer to think of themselves—along the lines that I have suggested here—as ‘in conversation’ with improvement researchers; or as gently ‘edging along’ the translational spectrum but maintaining a good distance from the most applied end of scholarship. Nonetheless it seems clear that there is a great deal of scope for scholars from many different disciplines to constructively contribute to improvement research, even if from a little distance, and not least by intensifying, extending and enriching the debates that are already happening within improvement research between advocates and sceptics of improvement research approaches.

## Combining the Tidy and the Unruly

The papers in this issue continue the kinds of ‘starter conversations’ that might be anticipated between dedicated improvement researchers and more diffuse contributors to thinking about better healthcare. They are written from diverse perspectives and have different foci but they share an orientation towards questioning assumptions about improvement. More specifically, as well as encouraging us to look in many disciplinary directions for inspiration, they invite us to be reflexive about the implicit normativity of quality improvement and to question both the epistemic and ethical norms that underpin both the practice and the study of healthcare improvement. This is the explicit concern of the concluding piece, by Stacy Carter, but it is also an organising theme of the Special Issue.

Trenholme Junghans illuminates the challenge by ‘digging deep’ into the conceptual metaphors and structures of feeling that inhabit improvement research. Her contribution—drawing upon anthropological and other lenses—shines a light on the picture of improvement science as about the ‘closing of gaps’ as symbolised by the IOM’s famous ‘quality chasm’—gaps, for example, between accepted best practice and what is often provided—in ways that suggest mechanistic imagery informed by engineering and statistical ways of thinking. Junghans exposes the risk that the underlying ‘scientistic’ habits of thought can act as a block to dealing with the complexities of improvement. However Junghans also draws attention to counter-currents which are alive within the improvement research community that reflect metaphors of complexity. This includes an account of quality improvement by the leading figures of Paul Batalden and Frank Davidoff that is positively open-ended and in which “Affective commitment and ceaseless striving become primary ends unto themselves, and improvement is cast as emergent, with no predefined limits or finite specifications.”

Junghans thus articulates and poses the problem that is central to any ambition to substantially broaden improvement research. How can we responsibly operationalise and measure improvement with sufficient exactness and rigour whilst at the same time embracing what she summarises as “the value of spaces of openness and ambiguity.” She is herself optimistic about the prospects of closing this particular gap but nonetheless the sense, and importance, of this gap is palpable in all of the other contributions.

The papers by Sheard and Reid are written at some distance from improvement research but with relevance to key dimensions of quality improvement—the role of both policy climates and of ‘human resources’ in shaping understandings and possibilities of healthcare improvement. Sally Sheard argues that historical analysis has the potential to improve both policy making and service delivery in healthcare whilst also demonstrating why this is far from an easy matter. She argues that the latter is first and foremost because history is often treated—albeit not necessarily self-consciously—as simply superfluous or irrelevant. This is a striking point of fundamental import—when we come together to think about healthcare improvement what modes of thought do not even get into the room, and what losses might this represent? History has the advantages of being geared towards complexity, of understanding how short-term changes are layered onto more recalcitrant longer-term structures and processes, and of allowing space for the imaginative exploration of counterfactuals. But its methods are relatively alien to those social and behavioural sciences that dominate the climates of improvement research and evidenced-based policy making. If history is to have influence in service delivery or policy making then there is ‘translational’ work to be done and Sheard illustrates what can and has been achieved to open up channels of communication and to make history practically applicable. She also reminds us that historians famously reject that form of weak thinking that represents forward movement as straightforward progress—so called Whiggish history. In reality change is typically complex and multivalent and it could be argued that if our accounts do not capture this—in history or in quality improvement—then we are simply looking at too few things from too few perspectives.

Lynette Reid considers the education of medical professionals as one foundation of good healthcare, focusing in particular on the way scientism shapes that foundation. Reid’s account starts by highlighting the persistence of the split and hierarchy between ‘hard’ and ‘soft’ knowledge in medical education—no matter how often it is acknowledged that medicine must transcend scientific modes of thought, scientism reasserts itself in new forms. In particular she underlines how the broadening out of the emphasis of medical education to include a range of competencies, including a concern with professionalism, has been accompanied by medical education scholarship that focuses on measuring the effectiveness and efficiency of content transmission. There is a need to divert the orientation of this mini research industry so as to ask critical questions about the context of medicine and the social problems that should determine, and the institutional pressures that arguably distort, medical priorities: “The decision to spend the funds of academic medicine on researching the efficiency of the educational process itself …rather than on developing expertise in safe, efficacious, and high-quality care and its organization and delivery is a substantial decision. It is not clear what warrants the present balance of funding for these two activities in academic medicine.”

In any case science, as properly understood, Reid argues should itself be more capacious and must involve ‘reflexive interrogation of our ways of knowing’. Her argument is that the social accountability movement in medicine can help us move beyond the science versus art dichotomy in medical education by directing our efforts to reforming the institutional relationships between academic medicine and the communities it serves. This entails overcoming some of the strong reductionist and individualistic currents in medical education and aiming to produce graduates who are motivated and equipped to work collaboratively with multiple stakeholders including citizens, and on the social (as well as the clinical) determinants of health, and who habitually approach quality through the frames of ‘relevance’ and ‘equity’ and not only cost-effectiveness.

The other two papers—by Constantina Papoulias and Stacy Carter—are written from vantage points close to healthcare quality improvement—and ask questions about how attempts to invite complexity and contestation into improvement research might work. Papoulias is wrestling with a central problematic of healthcare improvement: once we grant that what counts as a good quality service, or as a service improvement, depends upon ‘relevance’—that is, depends upon what matters to service users—then we are committed to taking both ‘patient experience’ and ‘patient voice’ seriously. But how can we ‘capture’ and mobilise these things, and can we really turn our hands to these tasks whilst maintaining the epistemic norms that prevail in mainstream improvement research? In her discussion of the use of photography and ‘participatory visual approaches’ to harness patients perspectives and agency Papoulias—in parallel to Junghans—is asking how, and how far, the tidy and the unruly faces of improvement research can be held together.

Amongst other approaches she discusses Experience Based Co-Design (EBCD) as a method of quality improvement. This is a well-evaluated and influential approach to incorporating patient experience and participation into service evaluation and improvement that utilises videos as one element of collaborative work between service stakeholders including patients and carers. This approach [along with its cousin, the Freirian inspired CPBR (Community Based Participatory Research)] deliberately brings both ‘bottom up’ perspectives and dialogical working into quality improvement. It thus fulfils the ethical function of engaging with patients and carers and doing so in ways that are constructive and action oriented, ensuring “that a direct and intensive engagement with patient experience is located at the very core of improvement efforts, thereby potentially challenging normative organisational habits of ‘doing improvement’.”

Nonetheless Papoulias worries that at least in some instances applications of EBCD risk ‘incorporating’ patients in what might be seen as a pejorative sense—potentially ‘taming’ their perspectives precisely in order to make them practically manageable. Whether or not this is a significant problem Papoulias argues (and shows, with reference to some of her own research) that the uses and analyses of participatory visual approaches have the potential to be disruptive and critically charged and beyond tidy incorporation. Her suggestion is that ‘photographs and film afford a different way of knowing, the benefits of which are vacated when images are used to simply bolster or emphasise textual or numerical evidence”, and that the most far reaching benefits of such methods might be to help us be reflexive about the prevailing ways in which ‘patient experience’—and indeed ‘empirical evidence’ itself—is constituted in our research.

Many of the themes reviewed here come to a head in Stacy Carter’s piece. Carter argues that there is a need to make the ‘implicit normativity’ of healthcare improvement research and practice more explicit. This means attending to, and debating, the values that permeate both the social field of healthcare and the business of healthcare improvement; she explains: “When I talk about implicit normativity, I am referring to the presence of unstated or taken-for-granted assumptions about what is good or bad, right or wrong, required or not required. When I propose that these should become more explicit, I mean that they should be openly acknowledged, and be justified by good reasons.”

All of the papers, albeit using different genres, highlight the dangers of wearing ‘blinkers’ in improvement research. Carter’s suggestion, crudely summarised, is that we should try to take the blinkers off. The very substantial challenge that this opens up however is that such a lot comes into view—when we look carefully it seems that disguised and often unexamined value judgements are everywhere to be seen. For anyone interested in normative issues this will not be surprising but it does mark a potentially big shift in focus for improvement research. Drawing on examples from cancer screening (and other areas) Carter works systematically through the constitutive role of values in agenda setting, the construction of evidence, definitions of health and disease, the organization and provision of services, approaches to patient and public engagement, and in citizens’ judgements about healthcare. Given this wealth of considerations surely the least we can do is to be reflexive about the epistemic and ethical norms that frame our approach to healthcare quality improvement. This is, in key respects, analogous to one of the contributions that the sociological work discussed earlier makes to the field—surfacing and disrupting normative assumptions.

I suggest that the approach Carter represents here lights the way ahead. However it also illuminates the extra complications that arise if we seek to take this path. It seems both that we are obliged to bring the consideration of normativity into improvement research and, at the same time, that it is far from clear how we might best do so. This is only in part about the generic, but deep-seated, concern that runs through all the issue as a whole—i.e. how to practically combine ‘tidy science’ with ‘unruly values’—it is also about different approaches to working with norms. Carter—as quoted above—stresses the importance of the norms that we rely on being ‘justified by good reasons’. In addition her account echoes other contributions by arguing for the central importance of harnessing the values of patients and publics as a way of grounding a more explicit approach to normativity.

The question I would want to raise is how these two ideas fit together? This is a question for a later date and is not meant as a question specifically for Carter but for all those of us who are interested in developing empirical bioethics as a field, including in its application to healthcare quality improvement. How—in principle and in practice—should we filter and weigh the manifold values that can inform both the purposes and processes of quality improvement? Clearly, for example, there are good reasons to take the values of citizens seriously—but how should these be treated relative to other normative judgements that we see as justified on other grounds? As signaled earlier it is one thing to assert—as I have—that improvement research must draw upon normative scholarship but it is quite another to show how that aspiration can be achieved in ways that also cross the ‘translational divide’.

The questions reviewed here, as I have said, only represent a small part of a much longer conversation. But, I believe, this Special Issue indicates that these continuing exchanges promise to be both intellectually and practically productive.
